# Risk of recurrence and bleeding in patients with cancer‐associated venous thromboembolism treated with rivaroxaban: A nationwide cohort study

**DOI:** 10.1002/cam4.1997

**Published:** 2019-02-14

**Authors:** Mette Søgaard, Peter Brønnum Nielsen, Flemming Skjøth, Jette Nordstrøm Kjældgaard, Torben Bjerregaard Larsen

**Affiliations:** ^1^ Department of Cardiology Aalborg University Hospital Aalborg Denmark; ^2^ Aalborg Thrombosis Research Unit, Department of Clinical Medicine, Faculty of Health Aalborg University Aalborg Denmark; ^3^ Unit for Clinical Biostatistics Aalborg University Hospital Aalborg Denmark

**Keywords:** anticoagulants, bleeding, cancer, rivaroxaban, venous thromboembolism

## Abstract

**Background:**

Rivaroxaban could be an attractive alternative to low molecular weight heparin for the treatment of cancer‐associated venous thromboembolism (VTE) but the safety and effectiveness remain unclear. We examined risk of recurrent VTE and major bleeding associated with rivaroxaban treatment of cancer‐associated VTE.

**Methods:**

Through linkage of nationwide Danish registries, we identified all adults with cancer‐associated VTE initiating treatment with rivaroxaban, 2012‐2017. We estimated rates and absolute risk of the primary outcome of recurrent VTE and major bleeding; all‐cause mortality was studied as a secondary outcome.

**Results:**

We identified 8901 patients with cancer‐associated VTE of whom 476 (5.3%) redeemed a prescription for rivaroxaban within 30 days of VTE diagnosis (mean age 71.5 years, 41% females, 57% with pulmonary embolism). Median time from cancer diagnosis to rivaroxaban prescription was 31 days (interquartile range 12‐73 days). Most frequent cancers were gastrointestinal (26.1%), genitourinary (23.3%), and hematological cancer (12.6%). Few had distant metastases (7.1%). At 6 months, recurrent VTE occurred in 6.1% (15.1 events per 100 person‐years) with the highest absolute risks for genitourinary cancer (8.1%), gastrointestinal cancer (7.3%), and breast cancer (6.5%). Major bleeding occurred in 1.9% (5.3 events per 100 person‐years), in particular, in genitourinary cancer (4.5%) and lung cancer (4.2%). Eighty deaths (17.8%) occurred during follow up.

**Conclusion:**

In this clinical practice setting, rivaroxaban was rarely used for cancer‐associated VTE. However, among those who received rivaroxaban, the treatment appeared safe and effective with rates comparable to previous studies of selected populations.

## 1 INTRODUCTION

Venous thromboembolism (VTE) is a frequent complication in patients with cancer.^1^ Oral anticoagulant (OAC) treatment is vital but also challenging because cancer can be associated with a hypercoagulable state, multi medication, invasive procedures, and increased risk of bleeding. As a consequence, both recurrent VTE and bleeding during treatment are more prevalent than in patients without cancer.^2^ Current clinical practice guidelines recommend treatment with low molecular weight heparin (LMWH) over vitamin K antagonists (VKA) or non‐VKA OAC (NOAC); this recommendation is well‐demonstrated with strong evidence for at least a 3‐month treatment duration (Grade 1A).^3^, ^4^, ^5^

For treatment of VTE in patients without cancer, the American College of Chest Physicians Evidence‐Based Clinical Practice Guidelines for antithrombotic therapy for VTE disease recently recommended NOACs including rivaroxaban as long‐term anticoagulant treatment (3 months or longer).^5^ The regulatory approval of the factor Xa inhibitor rivaroxaban for VTE was based on the EINSTEIN‐DVT and the EINSTEIN‐PE phase 3 noninferiority trials.^6^, ^7^ A pooled post‐hoc analysis of these trials indicated that compared with VKA, rivaroxaban was effective at reducing recurrent VTE and could be used for cancer patients without compromising safety.^8^ Recently, the results of the first two head‐to head comparisons between edoxaban and rivaroxaban, respectively, and LMWH also indicated that these regimens were comparable in terms of safety and effectiveness.^9^, ^10^

Rivaroxaban could be an attractive alternative to LMWH due to the once daily, oral dosing, which obviate the need for daily parenteral injections combined with short half‐life and lower price. Due to insufficient data examining outcomes in rivaroxaban and LMWH in cancer patients, we examined the safety and effectiveness of rivaroxaban in a nationwide cohort of patients with active cancer and VTE, and compared our findings with previous published studies reporting on treatment exposure and associated VTE recurrence in cancer patients.

## 2 MATERIAL AND METHODS

### 2.1 Setting and data sources

The source population for this cohort study comprised the entire population of Denmark encompassing 5.6 million inhabitants. Denmark has a tax‐funded universal health care system, with equal access to hospitals and primary care for all residents, and partial reimbursement of the costs of most prescribed medications, including OAC treatments. The health care system records most contacts with the health system, including births, deaths, hospital visits, and prescription claims.^11^ As a result, data on diagnoses and prescription claims are compiled in longitudinal national registries allowing a true nationwide population‐based study with no loss to follow up. This study was based on linkage of three nationwide registries; (a) the National Patient Register,^12^ including information from all inpatient stays and outpatient visits at Danish hospitals; (b) the National Prescription Register,^13^ which hold data on all prescription purchases by Danish residents since 1995; and (c) the Danish Person Registry,^14^ which contain data on sex, date of birth, vital, and emigration status. These registries were linked using the unique 10‐digit personal registration number assigned to each Danish citizen at birth and to residents upon immigration.^14^

### 2.2 Ethical considerations

The study was approved by the Danish Data Protection Agency (ref. 2015‐57‐0001). Ethical approval is not required for anonymous register‐based studies in Denmark. The data was provided by the Danish Health Data Authority.

### 2.3 Study population

We used the National Patient Register to identify all adult patients aged 18 years and older with active cancer and an inpatient or outpatient primary or secondary discharge diagnosis of VTE. As emergency room VTE diagnoses have low validity (positive predictive value of 31%), we did not consider these diagnoses.^15^ To ensure sufficient clinical record history for treatment and diagnoses, we excluded patients who had not been residents of Denmark for at least 1 year before the VTE diagnosis. Active cancer was defined as a diagnosis of cancer other than basal‐cell carcinoma of the skin, within 6 months of the VTE event, any treatment for cancer (see Table for codes) within the 6 months, or recurrent or metastatic cancer as described previously.^8^, ^16^ We classified cancers according to cancer site (breast, gastrointestinal, lung including pleura, genitourinary, gynecological, hematological, metastatic cancer, and other cancers) and cancer stage according to the TNM (Tumor, Node, Metastasis) classification categorized as localized, regional spread, distant spread, unstaged, and stage not recorded, (Table ).

Since we focused on outcomes under rivaroxaban exposure, patients were eligible for study inclusion; if they claimed any prescription for rivaroxaban within 30 days of discharge from the index VTE in the period from January 1, 2012 (rivaroxaban was introduced to the Danish market for recurrent VTE prophylaxis December 9, 2011) to December 31, 2017 (end of inclusion). We excluded patients with concomitant prescriptions for LMWH, warfarin, dabigatran, or apixaban within 30 days of the index VTE.

### 2.4 Study outcomes and comorbidity

We derived all primary outcomes from discharge diagnoses recorded in the National Patient Register (excluding emergency room diagnoses). The primary effectiveness outcome was the first recorded episode of recurrent VTE during follow up. Recurrent VTE was defined by a primary inpatient or outpatient diagnosis combined with an objectively confirmed VTE diagnosis using imaging in order to rule out repeated coding of the index event. According to a recent validation study, this ensures a positive predictive value of 82%.^17^ In addition, follow‐up began 10 days after the date of diagnosis of the index VTE to avoid repeated coding of the index event. The primary safety outcome was major bleeding events recorded as intracranial, gastrointestinal, and major bleeding in various anatomical positions and reported in total as “any bleeding.” Bleeding events were required to be primary inpatient diagnoses to increase the validity of the coded diagnosis. All‐cause death was included as a secondary outcome.

We ascertained comorbidities at baseline according to medication claims within the year before the index VTE event and/or history of primary or secondary hospital discharge diagnoses (excluding emergency room diagnoses) since 1994 (introduction of ICD‐10 in Denmark). Comorbidity information included cardiovascular and metabolic diseases, lifestyle‐related diseases, and indicators for surgery (Table ). We further combined covariate information into the Charlson Comorbidity Index^18^ (Table ).

### 2.5 Statistical analysis

Baseline characteristics at the time of index VTE diagnosis were described using means and standard deviations for continuous measures and percentages for categorical measures. Patients were followed until the outcome of interest, emigration, death, or end of study period (March 23, 2018), whichever came first. We used time‐to‐event analysis to analyze the risk of study outcomes at 6‐month follow‐up.

To enable comparison with other studies, we first calculated crude incidence rates as the number of events divided by person‐time. Then, we used the pseudovalue approach to estimate the cumulative incidence within 6‐month follow‐up, assuming death as competing risk.^19^ Allowing for a thorough evaluation, the main analysis was supplemented by analyses stratified by cancer site and cancer stage, and anticancer treatments within 30 days before the index VTE.

The analyses were performed using Stata/MP, version 15 (StataCorp) and R version 3.3.3 (The R Foundation).

### 2.6 Literature search methods

We searched PubMed Medline using Medical Subject Headings (MeSH) and free text to identify and summarize previous observational studies on the outcomes of rivaroxaban treatment in patients with active cancer and VTE. Development of the search strategy described in detail in Table was assisted by a trained medical librarian. All searches were performed at the end of December 2018. The first author reviewed the titles and abstracts and removed articles not relevant according to the population, intervention (exposure), comparison, and/or outcome (PICO criteria). The information summarized by our review was collected from the remaining articles and prior publications cited by these articles.

## 3 RESULTS

### 3.1 Nationwide cohort study in Denmark

**Figure 1:**
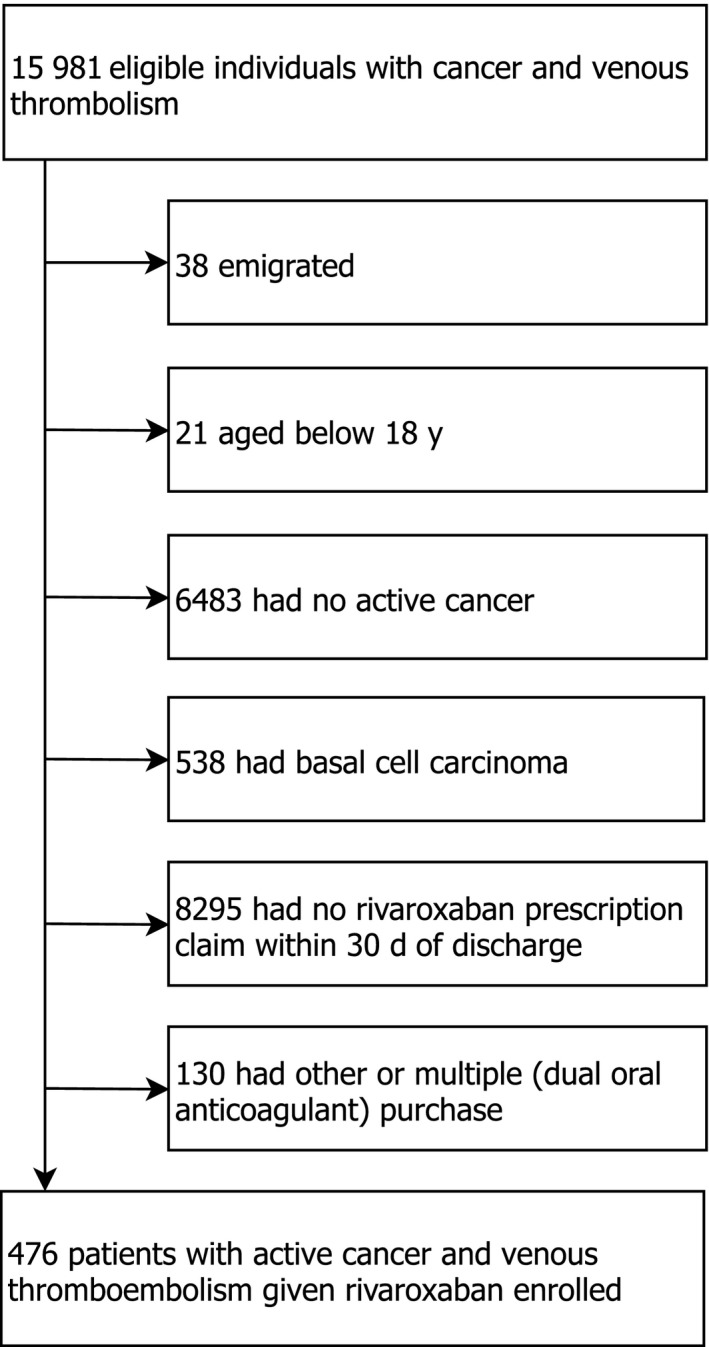
Flow chart of the study population

From January 1, 2012 to December 31, 2017, we identified 8901 patients aged 18 years or older with active cancer and VTE. Of these, 914 redeemed a prescription for rivaroxaban at any time following the index VTE; 744 initiated rivaroxaban within the first year and 476 within 30 days after the index VTE diagnosis. The final study population comprised these 476 patients with mean age 71.5 years (42% females, 57% with pulmonary embolism) (Figure 1, Figure ). Only 10 patients were diagnosed in 2012; the majority of patients was diagnosed in the latter part of the study period (Table 1). Median time from last cancer diagnosis to prescription redemption of rivaroxaban was 31 days (interquartile range, IQR, 12‐73 days); median time from the date of last anticancer treatment to prescription redemption was 67 days (IQR 28‐184 days) (Table 1). Forty‐nine patients (10.3%) had a previous VTE before cancer diagnosis and 8.2% were prior users of OACs. The most frequent cancer type was gastrointestinal cancer (26.1%) followed by genitourinary (23.3%) and hematological cancer (12.6%) (Table 1). Among the 406 patients with solid tumors, except for brain cancer, 7.1% had distant metastases. Cancer stage was unknown in 54.9% patients and not registered in 6.9%. Approximately one‐fifth (21.6%) had received anticancer treatment within 30 days before, in particular chemotherapy (15.5%) (Table 1).

**Table 1:** Patient characteristics

Characteristic	% (n)
N	476
Study year	
2012	2.1 (10)
2013	13.0 (62)
2014	15.8 (75)
2015	19.7 (94)
2016	25.4 (121)
2017	23.9 (114)
Female	42.0 (200)
Mean age (SD)	71.5 (10.8)
Cancer‐related characteristics	
Median time from last cancer diagnosis to first rivaroxaban prescription, days (IQR)	31.0 (12.0‐73.0)
Median time from last anticancer treatment to first rivaroxaban prescription, days (IQR)	66.5 (28.0‐184.0)
Any anticancer treatment with 30 d before VTE	21.6 (103)
Radiotherapy	5.9 (28)
Chemotherapy	15.5 (74)
Immune modulating, hormone or biological treatment	7.1 (34)
Cancer site	
Breast cancer	9.7 (46)
Gastrointestinal cancer	26.1 (124)
Lung cancer	10.1 (48)
Genitourinary cancer	23.3 (111)
Gynecological cancer	6.1 (29)
Hematological cancer	12.6 (60)
Metastatic cancer	2.1 (10)
Other cancer sites	10.1 (48)
Cancer stage for solid tumors except brain cancer^a^	
Localized	16.3 (66)
Regional	14.8 (60)
Distant	7.1 (29)
Unstaged	54.9 (223)
Stage not recorded	6.9 (28)
Medical history	
Prior VTE	10.3 (49)
Atrial fibrillation	8.4 (40)
Major surgery within 3 mo	36.8 (175)
Renal dysfunction	5.7 (27)
Alcohol‐related disease	5.3 (25)
Pneumonia within 3 mo	13.0 (62)
Vascular disease	11.1 (53)
Diabetes	12.8 (61)
Prior bleeding	14.3 (68)
Heart failure	10.1 (48)
Prior stroke	10.5 (50)
Chronic pulmonary disease	18.7 (89)
Myocardial infarction	4.6 (22)
Mean Charlson comorbidity index score (SD)	2.6 (2.4)
Concomitant drugs	
Prior oral anticoagulants	8.2 (39)
Systemic corticosteroids	18.7 (89)
Clopidogrel	5.9 (28)
Aspirin	22.1 (105)
Renin‐angiotensin inhibitor	35.7 (170)
NSAID	27.5 (131)
Statins	29.8 (142)
Loop diuretics	17.0 (81)
Non‐loop diuretics	28.6 (136)
Calcium channel blocker	18.9 (90)

SD; standard deviation, IQR, interquartile range; NSAID; nonsteroidal anti‐inflammatory drugs.

a 406 patients had solid tumors except brain cancer

**Figure 2:**
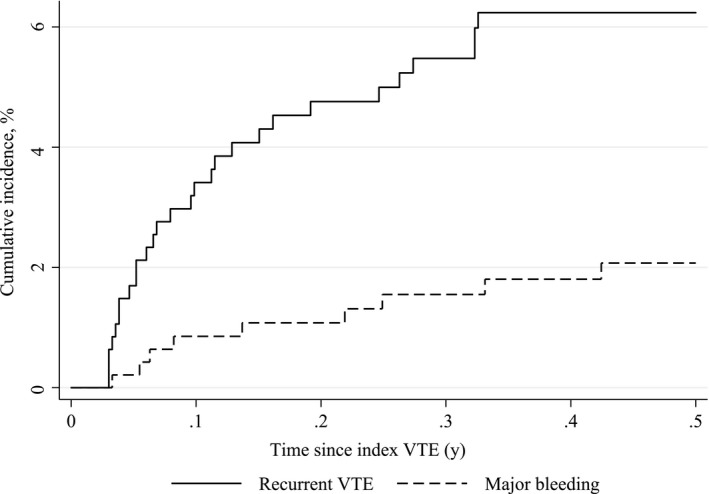
Cumulative risk of recurrent venous thromboembolism (VTE) and major bleeding for 6 months of follow‐up

Figure 2 displays cumulative incidence curves for recurrent VTE and major bleeding. As appears, most events occurred shortly after the index event. During 6 months follow‐up, 28 recurrent VTE events occurred (absolute risk 6.1%, rate of 15.1 events per 100 person‐years) with the highest absolute risk in patients with genitourinary cancer (8.1%), gastrointestinal cancer (7.3%), breast cancer (6.5%), and regional cancer stage (8.2%). Nine major bleed were diagnosed (absolute risk 1.9%, rate of 4.7 events per 100 person‐years), in particular, in genitourinary cancer (4.5%), lung cancer (4.2%), and patients with missing cancer stage (3.6%). Restriction to patients who received any anticancer treatment had little impact on the estimates (8.8% had a recurrent VTE, 1.0% experienced major bleeding). Among patients with chemotherapy treatment corresponding estimates were 6.8% and 1.4%, respectively.

Eighty deaths (17.8%) were occurred during 6 months follow‐up, equivalent to a rate of 40.6 events per 100 person‐years.

### 3.2 Literature review of previous observational studies

**Figure 3:**
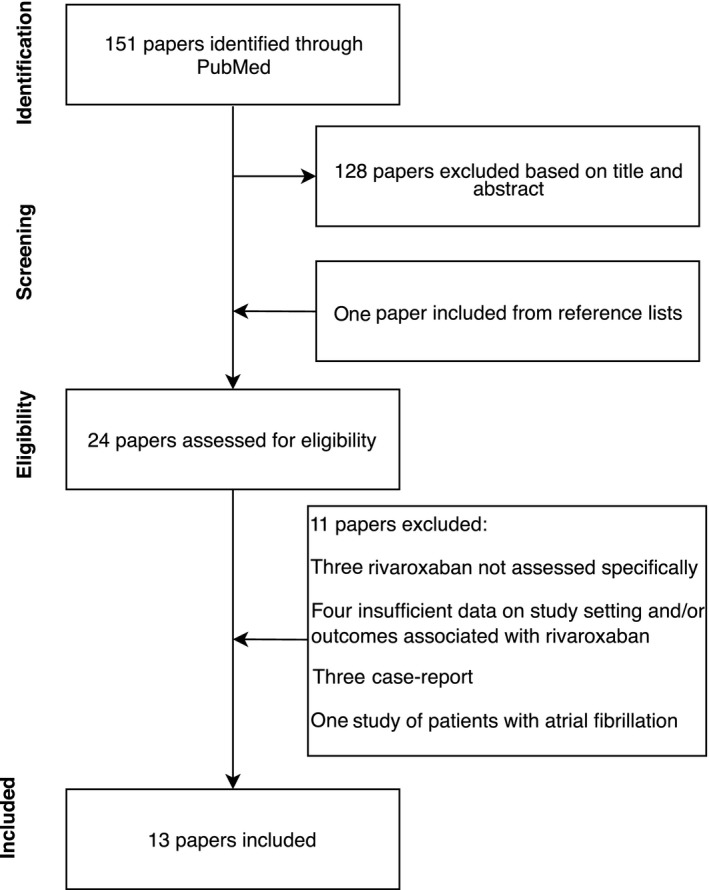
Flow diagram depicting the study selection process

Our initial search yielded 151 studies, of which 24 full‐text articles were retrieved and assessed for eligibility. After further exclusion of 11 studies, our review included 13 cohort studies (Figure 3). Study populations ranged from 41 cancer patients to 949, with 5 of the 12 studies including less than 100 patients. Two studies were restricted to patients with catheter‐related thrombosis and one study included only women with gynecological cancer. Duration of follow‐up varied across studies from 3 months to a mean follow‐up of 1.36 years. A detailed description of the studies is provided in Table .

Table 2 presents the absolute risk of VTE recurrence and major bleeding for our study compared with estimates retrieved by our review. The risk of recurrent VTE ranged from 0% to 13.2% with the most extreme estimates observed in studies including very few patients or not accounting competing risk of death leading to overestimation of event rates^20^, ^21^; in the majority of studies recurrence risk was approximately 3%‐5%. The risk of major bleeding ranged from 0% to 17%, again with the most extreme estimates obtained in studies with very few patients, for example the estimate of a 17% risk of major bleeding originated from studies with 18 and 48 patients, respectively.^22^, ^23^ Six studies provided estimates of clinically relevant nonmajor bleeding, which ranged from 1.2% to 12.2% (5 events) (Table 2; Table ). In addition to our study, eight studies provided data on all‐cause mortality which varied widely from 0.8% to 31.4% (Table 2).

**Table 2:** Event risk from observational studies on patients with active cancer and venous thromboembolism treated with rivaroxaban

Author, year	Follow‐up	Cancer patients with rivaroxaban, N	Recurrent VTE % (n)	Major bleeding % (n)	Clinically relevant nonmajor bleeding % (n)	All‐cause mortality % (n)
Søgaard et al, 2019 (present study)	6 mo	476	6.1% (28)	1.9% (9)	NA	17.8% (80)
Studies with 3 mo follow‐up						
Simmons et al,^31^ 2018	3 mo	98	1.0% (1)	5.1% (5)	6.6% (6)	4.1% (4)
Davies et al,^32^ 2017	3 mo	70	1.4% (1)	10% (7)	5.7% (4)	1.4% (1)
Laube et al,^33^ 2017	3 mo	83	3.6% (3)	2.4% (2)	NA	7.2% (6)
Nicklaus et al,^22^ 2017	3 mo	45	8.9% (NA)	17% (NA)	NA	NA
Studies with 6 mo follow‐up						
Yhim et al,^34^ 2018	6 mo	124	5.9% (7)	5.3% (6)	10.2% (11)	24.0% (28)
Kohn et al,^35^ 2018	6 mo	949	4.0% (37)	2.7% (22)	NA	11.3% (105)^a^
Streiff et al,^20^ 2018	6 mo	707	13.2% (119)	6.0% (63)	NA	NA
Chaudhury et al,^36^ 2017	6 mo	107	4.9% (3)	2.8% (3)	NA	NA
Mantha et al,^37^ 2017	6 mo	200	4.4% (8)	2.2% (4)	NA	17.6% (31)
Signorelli and Gandhi,^23^ 2019	6 mo	18	0	17% (2)	NA	NA
Studies with follow‐up>6 mo						
Bott‐Kitslaar et al,^38^ 2016	Mean follow‐up 1.36 ±0.5 y	118	3.4% (4)	2.5% (3)	3.4% (4)	31.4% (37)
Studies with unclear duration of follow‐up						
Wells et al,^39^ 2016	NA	237	3.8% (9)	1.3% (3)	1.2% (1)	0.8% (2)
Xavier et al,^40^ 2017	NA	41	12.2% (5)	None	12.2% (5)	NA

NA, not available

a Defined by in‐hospital deaths or hospice claims.

## 4 DISCUSSION

The findings of this nationwide cohort study of patients with active cancer and VTE indicate that use of rivaroxaban were safe and effective. The VTE recurrence rate of 6.1% and 1.9% major bleeds at 6 months follow‐up are in line with the estimates demonstrated by our review of previous cohort studies. Our findings also reveal that rivaroxaban was used infrequently in Danish cancer patients and presumably mostly for selected cancer patients. However, rivaroxaban was increasingly used during the study period.

### 4.1 Comparison with other studies

In 2014, rivaroxaban was prescribed in 20% of US patients with cancer despite lack of evidence and guideline recommendations to support this treatment.^24^ Sub‐group analyses of patients with cancer from large pivotal phase 3 trials have suggested that NOACs are noninferior to LMWH.^8^, ^25^ Yet, none of these trials were dedicated to cancer patients and less than 5% of the original trial populations had cancer. Considering that cancer patients typically represent 20% of all patients with VTE in the community, the included trial patients likely represent a highly selected subset of cancer patients encountered in routine clinical care. Nonetheless, based on available post‐hoc analyses of these RCTs and meta‐analyses, the updated International Initiative on Thrombosis and Cancer (ITAC‐CME) consensus statement recommendations from 2016 are that NOACs can be considered for VTE treatment of patients with stable cancer not receiving systemic anticancer therapy, and in cases where VKA is an acceptable, but not available, treatment choice.^3^

In 2017, results of the first two head‐to head comparisons between LMWH and NOAC for the initial and long‐term treatment of VTE in patients with cancer were presented.^9^, ^10^ The Hokusai VTE cancer trial randomized 1050 cancer patients with acute VTE to edoxaban or LMWH.^9^ Over 12 months of therapy, recurrent VTE developed in 7.9% in the edoxaban group and in 11.3% in the LMWH group; major bleeding complications occurred in 6.9% vs 4.0%. The excess bleeding events in the edoxaban group were mainly driven by upper gastrointestinal bleeds in patients with gastrointestinal cancer. The 2‐phase pilot trial SELECT‐D, which randomized 406 cancer patients to rivaroxaban and LMWH reported a 6 months recurrence rate of 4% in the rivaroxaban group and 11% in LMWH group; rates of major bleeding were 4% vs 3%, and there were substantially more clinically relevant nonmajor bleeds with rivaroxaban than LMWH (13% vs 2%).^10^ Several other trials are ongoing.^3^

Limited data are available on the selection and uses of rivaroxaban in clinical practice outside clinical trials, and it is debatable whether results from carefully selected trial populations can be extrapolated to routine clinical care. Different factors, patient‐related or based on a clinical decision, can affect the preferred treatment. The present study therefore adds to the existing knowledge. The risk of recurrent VTE in the present study is in line with the estimates of previous trials whereas the risk of bleeding was substantially lower and no excess bleeding events in gastrointestinal cancers were observed. This indicates that in selected cancer patients with VTE encountered in routine care, rivaroxaban may be a safe and effective alternative to LMWH. Taken together with the findings of our review, cumulative evidence suggests that rivaroxaban could be a favorable alternative to LMWH in selected cancer patients. However, the output and insights from the ongoing RCTs should confirm our observations before strong treatment recommendations can be presented.

Cancer patients with VTE is a heterogeneous and complex group of patients. Nonetheless, current practice recommendations apply to all cancer patients, irrespective of cancer site, spread, and anticancer treatments. It is very likely that the biological and clinical heterogeneity influence thrombogenicity and response to treatment. Treatment with LMWH can be burdensome for the patients because the treatment requires daily subcutaneous injections. The need for once or twice daily injection with LMWH might be acceptable for cancer patients but oral alternatives would undoubtedly increase quality of life and prevent post‐injection subcutaneous fibrosis.^26^ In a recent cohort study the cumulative 6‐month incidence of LMWH discontinuation among patients with cancer‐associated VTE was approximately 20%.^27^ The most common reason for discontinuation was pain at the injection site, followed by injection site hematoma and allergic reactions.^27^ Rivaroxaban is administered orally once daily and the short half‐life (5‐9 hours)^28^ facilitate temporary interruptions for procedures or periods of thrombocytopenia. However, despite practical advantages of rivaroxaban, the use may be limited by the potential increased risk of gastrointestinal bleeding especially in gastrointestinal cancers. An antidote to immediately reverse the action in case of bleeding has been tested in a phase 3 trial but is not currently on the market.^29^ Furthermore, there is a risk of drug interactions with anticancer treatments, especially those that interacts with P‐glycoprotein and cytochrome P450 3A4.^3^ Whether these interactions are clinically important remain unclear. In this study, event rates were not notably different among patients with recent chemotherapy or other anticancer treatments. It is likely that more profound knowledge and understanding of various anticoagulant options would allow clinicians to individualize management according to primary tumor site, anticancer treatment, concomitant medications, and interventions and thereby enhance patient outcomes and quality of care.

### 4.2 Strengths and limitations

Strengths of this study include data from a nationwide real‐world population with access to free health care, which is not subject to the selection biases that could affect the previous observational studies that included only subsets of cancer patients. All data were collected prospectively within the nationwide registries, which have full coverage for hospital admissions, outpatient care visits, and filled prescriptions with no patients lost for follow‐up.

This study also has limitations. The lack of a comparison group limits the interpretation of our results. We had no data on in‐hospital treatments; therefore, we were unable to identify a cohort of LMWH users since in Denmark, this treatment in cancer patients is generally administered by the hospitals and not recorded in the prescription database. Using VKA users as comparisons are problematic because this treatment is not the guideline recommended treatment and has been demonstrated as less effective than LMWH in cancer patients despite maintenance of INR within therapeutic range.^30^ Another limitation is our reliance on prescription purchase as proxy for medication usage, since patients might not take their anticoagulant drug.

## 5 CONCLUSION

In conclusion, this nationwide cohort study revealed that rivaroxaban was rarely but increasingly used to treat VTE in patients with active cancer in routine clinical practice in Denmark. In patients selected to this treatment, rivaroxaban appeared safe and effective with rates comparable to what has been reported in randomized trials and in minor observational studies based on selected patient populations. Additional studies are needed to delineate what types of cancer patients that might benefit from NOAC treatment.

## CONFLICTS OF INTERESTS

Associate Professor Larsen has served as an investigator for Janssen Scientific Affairs, LLC and Boehringer Ingelheim, and has been on the speaker bureaus for Bayer, BMS/Pfizer, Roche Diagnostics, Boehringer Ingelheim and Takeda Pharma. Flemming Skjøth has been consultant for Bayer. Peter Brønnum Nielsen has received speaking fees from Boehringer Ingelheim, consulting fees from Bayer; and grant support from BMS/Pfizer. Other authors – none declared.
